# Prof. P. F. Richter on Some New Ideas on Diseases of Metabolism

**Published:** 1908-08-01

**Authors:** 


### PROF. P. F. RICHTER ON SOME NEW IDEAS ON DISEASES OF METABOLISM.

Translation of paper read before Anglo-American Medical Association in Berlin. Specially reported for The Hospital.

Recent investigations on metabolism have shown how intimate the relations are between laboratory work and the clinic. Of course I shall only deal here with subjects which are of actual clinical significance, so I wish to emphasise that recent research has proved how diseases of metabolism like gout, diabetes, and adiposity are related to one another. It is true from practice we know how much these diseases are related in the nosologic system, but why this is so we only know from the researches of the last decade. Formerly one used to say that a retardation of metabolism took place in these diseases. But this is not exactly correct: it is not a retardation, but an insufficiency of the organism to transform definite substances.

In diabetes, for instance, we have a disturbance of metabolism of sugar, which the organism is unable to transform. Now we know that adiposity and gout show similar disturbances of metabolism; in the former the organism is insufficient for fat, in the latter for uric acid. Uric acid produced in the body is not burned up as in a normal organism. Insufficient oxidation occurs in all these diseases.

In obesity the chief thing that has been pointed out of late years is that an obese diathesis does indeed exist. Formerly one explained this disease as being a malproportion between the ingestion and the use of fat in the organism. If the individual took too much fat, more than he could digest, he increased his adipose tissue. This is true, but at the same time, it is not a disease. But there are, indeed rare cases of constitutional obesity, and we have to ask, why do such persons become fat? Our answer is now, not only that oxidation of fat in the cell is disordered, but that the organism is unable to oxidate fat at all. This has been demonstrated by experiments on the gas-production of the organism. The obesity of women after splaying or after the climacteric likewise shows that there is indeed a constitutional obesity. In these cases the organism is deprived of a substance necessary for the oxidation of fat. If we administer ovarian extract to splayed animals, the slow metabolism of fat will be increased. This shows that previously a secretion for the oxidation of fat was lacking. Fortunately these cases are comparatively rare. I say fortunately, for there are but few means of increasing the metabolism of fat. The most effective one is ovarian extract, but only in women after the climacteric. Thyreoidin is a very dangerous remedy, oxidation is, indeed, increased thereby, but oxidation of albumen goes on at the same time. By prolonged use of thyreoidin we may cause anæmia and nervous conditions as a sequela of the intoxication. Boracic acid is another, but less dangerous, remedy. It helps, indeed, to reduce the weight of the body, but it is not very effective. According to the latest researches diet is the best means of reducing the weight of the body. The main factor in the modern therapy of obesity is the qualitative reduction of the food.

Now there has been for a long time a discussion as to whether, and how, one should reduce the food qualitatively. All are agreed on one point, albumen should not be reduced. Indeed a good cure for obesity must always spare the albumen of the organism, and must only reduce the ballast. As to the question whether one should withdraw carbohydrates or fat, I think this is a matter for experiment. As a rule, the chief thing is to reduce the amount of fat, but not to withdraw carbohydrates. For in the first place the latter make the patient feel satisfied, and in the second the carbohydrate foods contain albumen.

Drinking also is now considered from quite a different standpoint. We do not think nowadays that a thirst-cure is necessary in the therapy of obesity. Ortel and Schwenniger are not right, if they regard it as indispensable to restrict the quantity of water drink. This is only justified in cardiac insufficiency. In simple obesity the reduction of water may even do harm. Since the tissues of people suffering from obesity are poor in water, we shall produce by the reduction of drinks a tendency to calculus of the gall-bladder and of the kidney. Therefore we shall allow, on the contrary, any amount of drinking.

The principal points of my therapy for obesity are:—

1. Albumen, as usual, about 100 gr. albumen per day.
2. No fat, no butter, no cream, milk or sauces.
3. Potatoes are allowed, if given without fat. The pure potato is no fat-forming substance. I prefer the potato cure, and allow vegetables too. These carbohydrates are poor in fat and calorics. I am used to give for dinner 200 gr. potatoes (equal to five to six potatoes), and 150 gr. potatoes in the evening. Between, I give vegetables containing much cellulose, as radishes. By this food the patients are easily satisfied, and lose in weight, but at the same time are not losing albumen. The patients are allowed to drink as much as they like, especially mineral water; of course no alcohol. Drinking ten minutes before the meals will help to make the patients feel satisfied.

### Diabetes and Gout.

The therapy of diabetes may unfortunately be dealt with briefly. We have not made great progress in this direction, and have not found a specific remedy to restore the disordered oxidation of sugar. Even the organotherapy with pancreatic extract has proved ineffective. This is quite obvious, for we know that the secretion of the pancreas is only the activator of the various glycolytic ferments. Scientific therapy is therefore not yet possible, since we have not yet succeeded in finding the glycolytic ferments. The only thing we can do is to combat the acidosis, the forerunner of coma. Formerly we administered carbohydrates, and indeed laevulose is very effective, especially as a prophylactic. This sugar is very easily oxidised by the diabetic organism. In addition to laevulose I can recommend alcohol (cognac) in big doses. Alcohol diminishes the production of acetone. But in order to neutralise the acetone, we must also administer an alkali, at least 25-30 gr. daily, or the production of acids is very great in the diabetic organism. You have to begin very early with the cure. As soon as acetone is present, give alkali subcutaneously or per os.

Finally, some words on gout, a disease on which most research has been carried out. For therapeutics, it is true, we cannot draw many conclusions from these investigations. But the chief result is that confidence in the old uric acid theory is renewed. All the investigations have proved that uric acid produces gouty deposits. One can produce these foci by injection, or feeding animals with uric acid. The accumulation of uric acid in the organism is due to a deficient oxidation of uric acid in the body. The liver, the muscles, and the kidneys have ferments which break down uric acid. These ferments are absent in gout. We have therefore, as in diabetes, a disorder of the metabolism of urea. This must be the basis of therapy. We can say nowadays with certainty that the purin bodies have to be withdrawn from the organism. Since the blood contains too much uric acid, we have to exclude substances which produce uric acid. Now the question is often discussed whether we could not improve the solubility of uric acid; a long time ago alkalines were recommended for the treatment, but modern researches have shown that alkalines do not increase the solution of uric acid, but rather diminish it. It is further remarkable that after the injection of alkali the deposit of uric acid is even increased. Modern authors therefore consider the hydrochloric acid therapy the best. Falkenstein has stated that in gout acidity exists in the stomach. This is, however, not true. I cannot obtain good results by the hydrochloric acid treatment, and I think we shall do better to keep to the old alkaline therapy. You see, therefore, there are certain contrasts between the clinic and the laboratory, which require further investigations.

